# A retrospective analysis for investigating the relationship between FIGO stage IVA/IVB and cytoreductive surgery with prognosis in epithelial ovarian cancer

**DOI:** 10.3389/fonc.2023.1103357

**Published:** 2023-07-24

**Authors:** Hong Liu, Min Luo, Chunrong Peng, Jianmei Huang, Dengfeng Wang, Jianming Huang, Guonan Zhang

**Affiliations:** ^1^ Gynecologic Oncology Center, Sichuan Clinical Research Center for Cancer, Sichuan Cancer Hospital & Institute, Sichuan Cancer Center, Affiliated Cancer Hospital of University of Electronic Science and Technology of China, Chengdu, China; ^2^ Department of Biochemistry & Molecular Biology, Sichuan Clinical Research Center for Cancer, Sichuan Cancer Hospital & Institute, Sichuan Cancer Center, Affiliated Cancer Hospital of University of Electronic Science and Technology of China, Chengdu, China

**Keywords:** ovarian cancer, FIGO stage IV, diagnosis, therapy, prognosis

## Abstract

**Objective:**

To investigate the effect of primary debulking surgery (PDS), NACT followed by interval debulking surgery (NACT-IDS), and chemotherapy alone on the prognosis of FIGO stage IV epithelial ovarian cancer (EOC) with different metastatic patterns.

**Methods:**

We retrospectively analyzed 133 cases of FIGO stage IV EOC with pleural effusion (stage IVA), parenchymal metastases (stage IVB), or extra-abdominal lymph node metastases (stage IVB) at our Hospital between January 2014 and July 2021.

**Results:**

Among 133 cases with stage IV disease, 16.5% (n=22) presented with pleural effusion, 46.6% (n=62) with parenchymal metastases, and 36.9% (n=49) with extra-abdominal lymph node metastases. Regardless of the metastatic patterns, the 90.2% (n=120) of cases who underwent PDS/NACT-IDS exhibited a significantly superior overall survival (OS) compared to the 9.8% cases (n=13) who received chemotherapy alone (32 vs 17 months, *p*=0.000). The cohort was further stratified into 58 cases (48.3%) with R0, 41 cases (34.2%) with R1, and 21 cases (17.5%) with R2. The median OS of cases with R0 was significantly better than that of cases with R1/R2 (74 vs 27 months, *p*=0.000). There was no significant difference in median OS between PDS and NACT-IDS (43 vs 31 months, *p*=0.676), as well as between FIGO IVA and IVB (35 vs 31 months, *p*=0.582). Additionally, the metastatic patterns and the number of neoadjuvant chemotherapy cycles (≤4 or >4) did not demonstrate any prognostic significance for median OS (*p*=0.820 and 33 vs 26 months, *p*=0.280, respectively).

**Conclusion:**

Regardless of FIGO IVA and IVB stages or metastatic patterns, patients diagnosed with stage IV EOC may benefit from cytoreductive surgery with abdominal R0, compared with chemotherapy alone.

## Introduction

Epithelial ovarian cancer (EOC) is the eighth leading cause of death in women worldwide. The latest global cancer data show that EOC burden rises to 31 0,000 new cases and 21 0,000 deaths among women worldwide in 2020 (1). In the majority (up to 70%) of EOC patients, the disease is diagnosed in an advanced stage, disseminated intra- and/or extra abdominally. Stage IV ovarian cancer (FIGO 2014), defined as tumor spread outside the abdominal cavity (malignant pleural effusion, stage IVA) and/or (parenchymal metastases or extra-abdominal lymph node metastasis, stage IVB) is present in 12-33% of the patients at initial diagnosis (2–4). Overall, median survival for patients with stage IV disease ranges from 15 to 29 months, with an estimated 5-year survival of approximately 20% (5, 6). Stage IV patients’ 5-year survival rates were 8% and 39% with suboptimal and optimal debulking surgery (5, 7), respectively, because their survival is predominantly influenced by the residual tumor load and the response to chemotherapy.

The current standard treatment is primary debulking surgery (PDS) followed by six courses of platinum-based chemotherapy or neoadjuvant chemotherapy followed by interval debulking surgery (NACT-IDS) for FIGO stage IV EOC. Nonetheless, it is debated whether women with FIGO stage IV EOC should be offered PDS or NACT-IDS (5, 8, 9). and the impact of complete resection of intra-abdominal disease (R0) despite their extra-abdominal metastases is controversial (10–12). Moreover, there is limited data on the prognostic significance of the sub-classification of stage IV EOC (IVA and IVB). Therefore, more needs to be done to advance the understanding of the optimum course of treatment aiming at the complete cytoreduction of all macroscopically visible diseases for patients with stage IV EOC.

In this study, we investigated the impact of intra-abdominal residual diseases, FIGO IVA vs IVB, the pleural effusion, parenchymal metastases, or extra-abdominal lymph node metastases defining stage IV disease on overall survival (OS) comparing PDS, NACT-IDS, and chemotherapy in FIGO stage IV EOC.

## Materials and methods

### Patients

Data of patients with epithelial ovarian cancer (EOC) were extracted from Sichuan Cancer Hospital Database. We included 133 EOC patients with FIGO stage IV (the 2014 FIGO staging system) from January 2014 to July 2021 in our hospital. Follow-up started from the date of EOC diagnosis to the date of death, last follow-up, or July 31, 2022.

Patient characteristic information, including age, histologic type, ascites condition, serum CA125 level, patterns of metastasis at initial diagnosis, treatment, postoperative residual size of abdominal disease and first recurrence site, etc. were collected. Metastatic adenocarcinoma of unknown origin, borderline tumor, concomitant other malignant disease, and non-epithelial histology were excluded in this study. All patients were evaluated with ‘Suidan’ criteria (age, ASA score, CA125, CT results) for primary debulking (13). Among EOC patients included in this study, those with predictive score ≥ 3 before surgery and uncertain R0 tumor resection for PDS received NACT, those with response to NACT received IDS, and those with an inoperable tumor and progresses during NACT received chemotherapy only. In addition, all tumors, and isolated parenchymal and lymph node metastases in EOC patients were resected completely without macroscopic visible residual disease via cytoreductive surgeries at least including total hysterectomy, bilateral salpingo-oophorectomy, total omentectomy, and appendicectomy +/− pelvic and para-aortic lymphadenectomy.

All patients at initial treatment received platinum based intravenous chemotherapy. Among them, 108 patients received paclitaxel (135–175 mg/m^2^) plus carboplatin (AUC 5-6), 25 patients received docetaxel (60-70 mg/m^2^) plus carboplatin (AUC 5-6).Patients with platinum sensitivity relapse received paclitaxel/carboplatin, gemcitabine/carboplatin, liposome doxorubicin/carboplatin, docetaxel/carboplatin, and in patients with resistant or refractory relapse (PFI<6 months) received monotherapy with a non-platinum drug, including liposome doxorubicin, albumin paclitaxel, irinotecan, isocyclic phosphoramide, topotecan and oxaliplatin.

FIGO stage IV disease was defined as: localization of metastases, pleural malignant effusion (stage IVA) with positive cytology, parenchymal metastases (either intra-abdominal or extra-abdominal), or extra-abdominal lymph node metastasis (stage IVB), cytologically and/or histologically verified, or diagnosed by imaging alone. Data regarding stage IVA and IVB, localization of metastases, method of diagnosis, and possible reason for refraining from surgery were obtained or validated through patient records.

### Statistical analysis

Clinicopathological factors were analyzed by χ^2^ test or Fisher’s exact test for categorical variables. Survival analysis was performed using the Kaplan-Meier method or Cox proportional hazard models. Overall survival (OS) was defined as the interval ranging from the date of the primary surgery or the first cycle of neoadjuvant chemotherapy to the date of death or the last follow-up. All analyses were performed using SPSS version 27.0 (IBM Corp., Armonk, NY, USA), with *P*<0.05 considered to be significant.

## Results

### Patients and clinicopathological features

The clinical features of 133 patients are shown in (**Table 1**). The median age at diagnosis was 53 years (30~75 years) and the World Health Organization performance status (WHO PS) was 0 to 1. Histologic subtype was as follows: 126 cases of high-grade serous adenocarcinoma, 5 cases of clear cell carcinoma (among them, 3 cases received PDS followed by chemotherapy with paclitaxel/carboplatin for 6-8 cycles, 2 cases received IDS after neoadjuvant chemotherapy with paclitaxel/carboplatin for 3-4 cycles, and then continued chemotherapy with paclitaxel and carboplatin for 4-5 cycles). 2 cases of low-grade serous adenocarcinoma received PDS followed by chemotherapy with paclitaxel/carboplatin for 6 cycles. Among 133 cases, 22 cases (16.5%) were stage IVA with pleural effusion and 62 cases (46.6%) with parenchymal metastases (among them, there were 20 cases with liver metastasis, 11 cases with gastrointestinal tract metastasis, 2 cases with abdominal wall puncture hole metastasis, 1 case with bone metastasis, 8 cases with lung metastasis, 4 cases with pleural metastasis, 1 case with spleen metastasis, 15 cases with multiple site metastasis) and 49 cases (46.6%) with extra‐abdominal lymph node metastases (among them, there were 5 cases with mediastinum node metastasis, 2 cases with axillary node metastasis, 12 cases with cervical lymph nodes node metastasis, 4 cases with inguinal lymph nodes node metastasis, 5 cases with supraclavicular lymph nodes node metastasis, 7 cases with cardiophrenic angle lymph nodes metastasis, 14 cases with multiple site lymph node metastasis) were stage IVB. 19 cases (14.3%) underwent PDS, 101 cases (75.9%), underwent NACT-IDS, 13 cases (9.8%) with comorbidities were considered unresectable and poorer response to chemotherapy.

**Table 1:** Patient characteristics.

Parameters	N (%)
Age, median (range)	53 (30-75 ys)
FIGO stage	
IVA	22(16.5%)
IVB	111(83.5%)
Patterns of metastasis	
Pleural effusion	22(16.5%)
Extra‐abdominal lymph node metastases	49(36.9%)
Parenchymal metastases	62(46.6%)
CA125 (U/mL)	
<500	34(25.6%)
≥500	99(74.4%)
Ascites	
No	25(18.8%)
<500 mL	47(35.3%)
≥500 mL	61(45.9%)
Residual disease	
R0	58(48.3%)
R1	41(34.2%)
R2	21(17.5%)
Treatment	
IDS	101(75.9%)
PDS	19(14.3%)
Chemotherapy only	13(9.8%)
Histologic type	
High grade serous	126(94.7%)
Other	7(5.3%)
NACT cycles (IDS)	
≤4	76(75.2%)
>4	25(24.8%)

NACT, neoadjuvant chemotherapy; IDS, interval debulking surgery.

### Rate of R0 resection for cytoreductive surgery

Following cytoreductive surgery, 120 cases (90.2%) were evaluated for complete resection (R0, no macroscopic visible intra-abdominal residual disease) or (R1, residual tumor >0 and ≤1 cm) or (R2, residual tumor >1 cm). Among them, there were 58 cases (48.3%) with R0, 41 cases (34.2%) with R1, and 21 cases (17.5%) with R2; the rates of R0 resection in 6 of 19 PDS patients (15.8%) and 52 of 101 NACT-IDS patients (84.2%) were 31.6% and 51.5%, respectively, no significant difference was shown between them (χ^2^ = 2.538, *p*=0.111).

### Recurrence of platinum resistance

The platinum-resistant relapse is defined as disease progression within 6 months after the last platinum-based chemotherapy. In terms of incidence of platinum resistant recurrence, the relapse rate was 6 (40%) of 15 patients for PDS compared to 50 (72.5%) of 69 patients for NACT-IDS (χ^2^ = 5.843, *p*=0.016). Among 101 cases for NACT-IDS, 76 (75.2%) received ≤ 4 cycles of NACT followed by early IDS and 25 (24.8%) received >4 cycles of NACT before delayed IDS. In 69 cases with relapse of platinum resistance for NACT-IDS, the relapse rate was 36 (72%) of 50 patients who received ≤4 cycles of NACT compared to 14 (73.7%) of 19 patients who received >4 cycles of NACT (χ^2^ = 0.020, *p*=0.889).

### Survival

**Figure 1:**
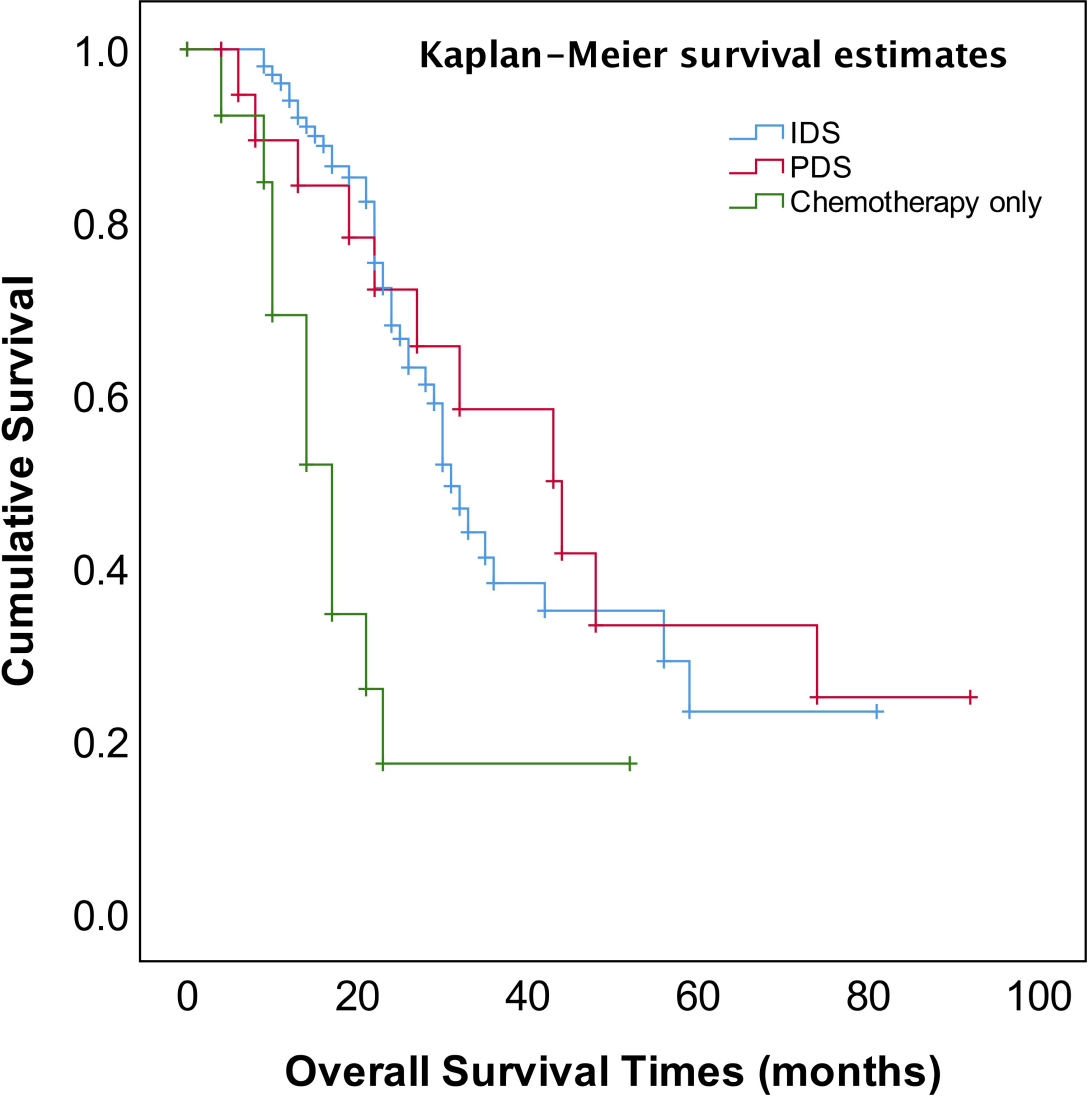
OS in patients with stage IV EOC for PDS, NACT-IDS, and Chemotherapy only.

The median follow-up time was 23 months (range, 4~92 months). The patients for PDS or NACT–IDS had better overall survival (OS) compared to those for chemotherapy only (*p*=0.001) (**Figure 1**). The median OS of patients for cytoreductive surgery was 32 months compared to 17 months of those for chemotherapy only (*p*=0.000). However, there was no significant difference in median OS between PDS and NACT-IDS (43 vs 31 months, *p*=0.676). Also, the median OS of patients receiving ≤4 NACT followed by early IDS was not statistically superior to that of those receiving >4 cycles NACT followed by delayed IDS (33 vs 26 months, *p*=0.272).

**Figure 2:**
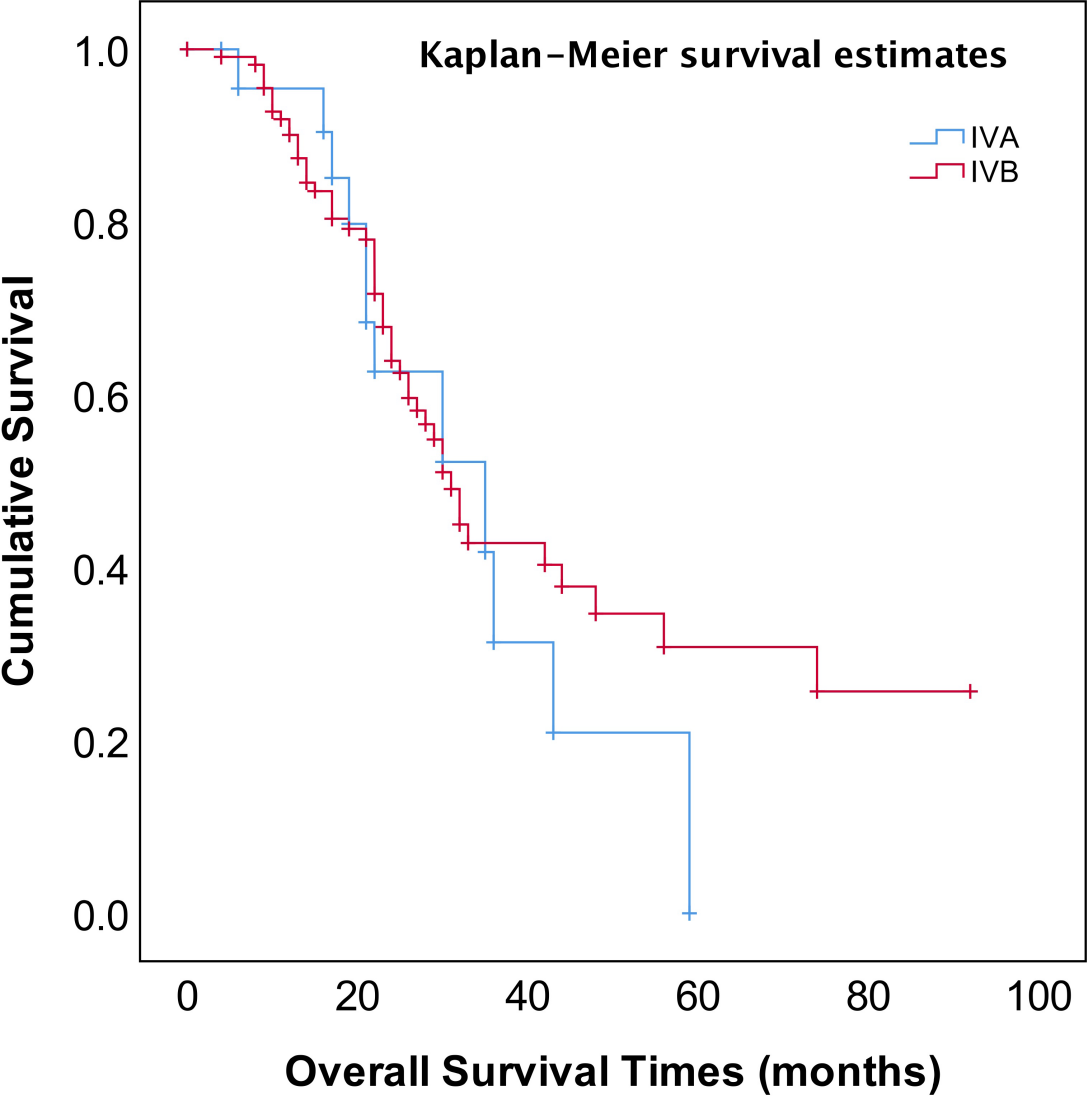
OS in patients with stage IV EOC with FIGO IVA or FIGO IVB.

**Figure 3:**
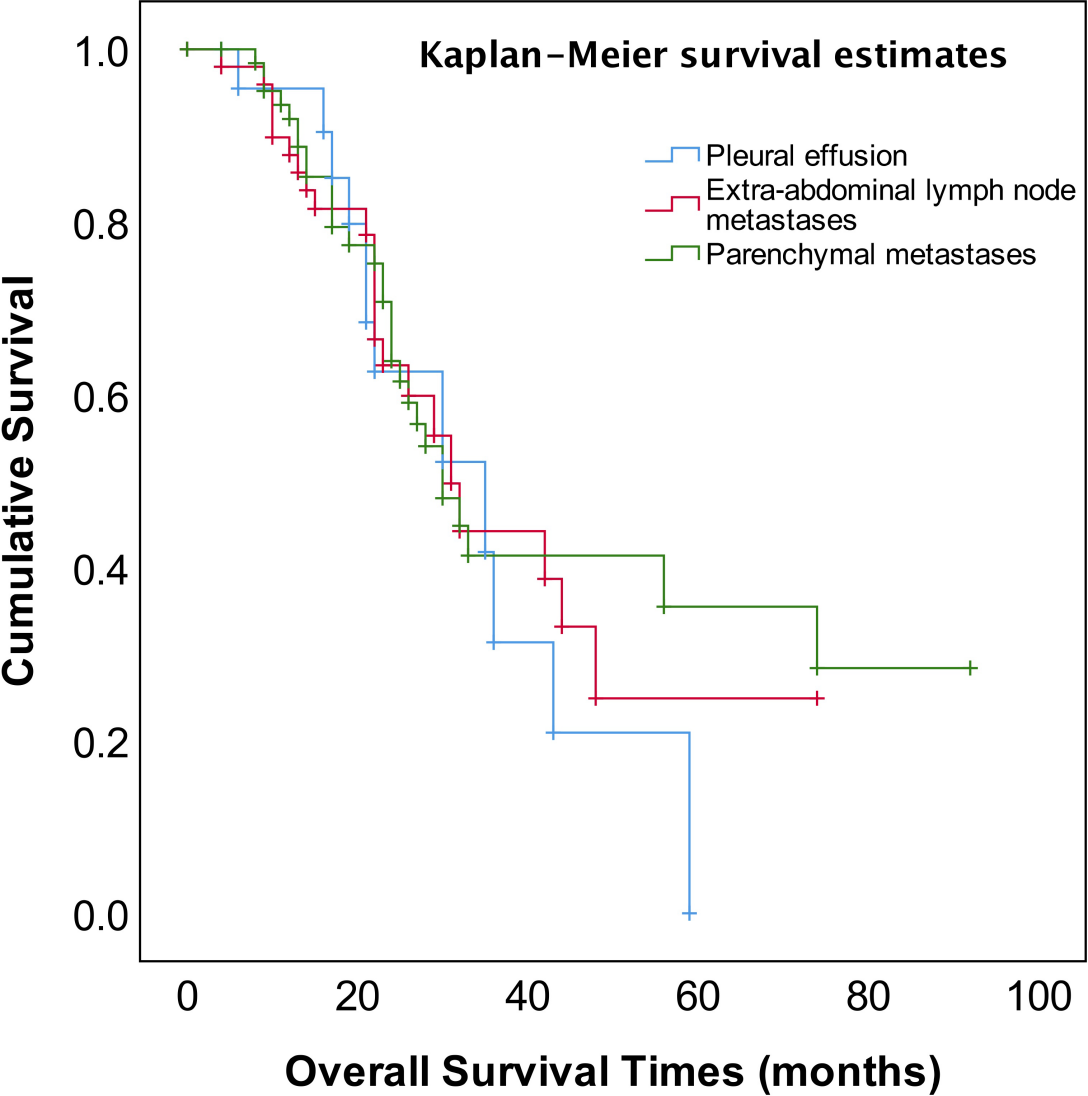
OS in patients with stage IV EOC with three patterns of metastatic disease.

**Figure 4:**
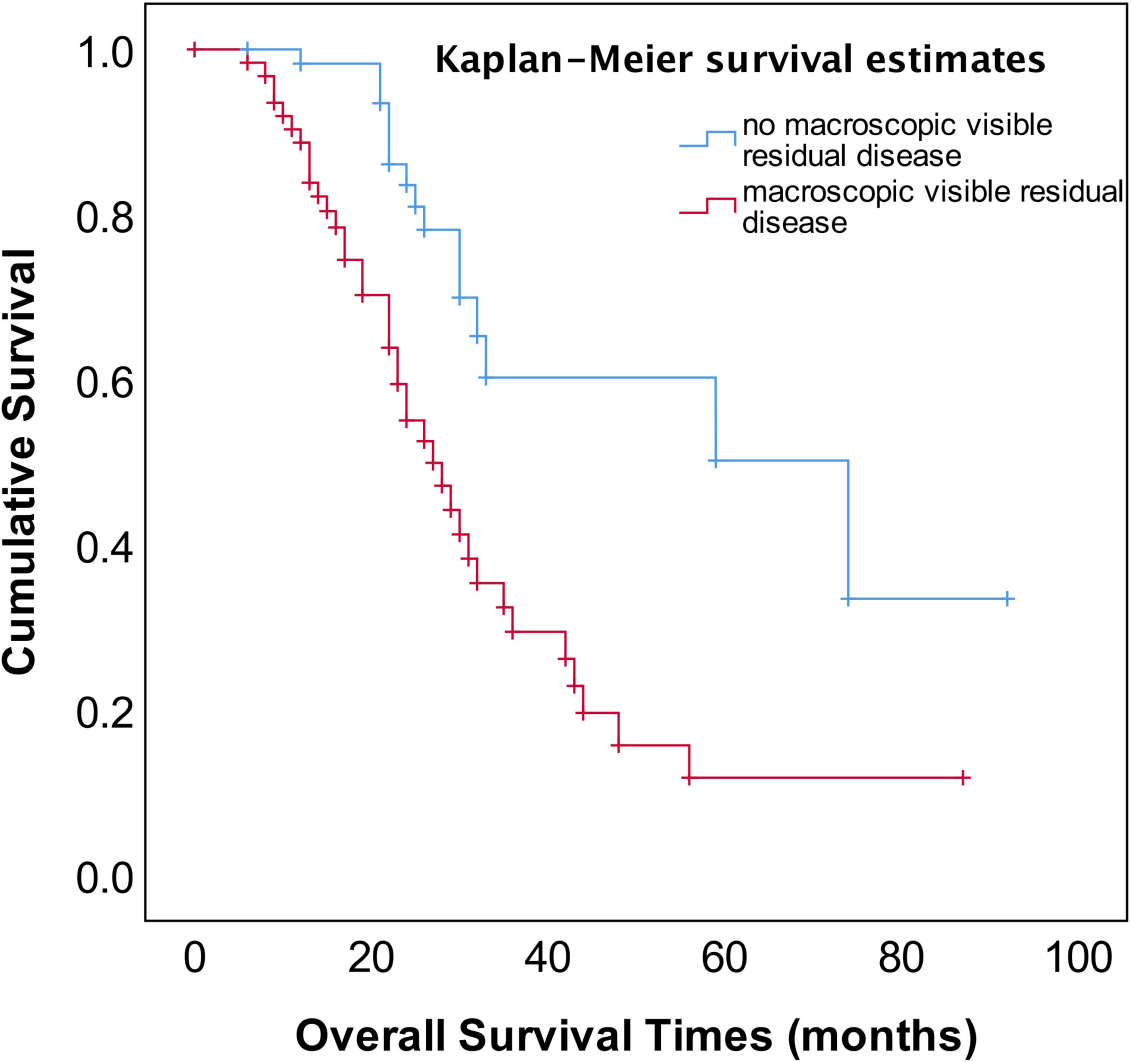
OS in patients with stage IV EOC with stratified status according to extent of abdominal residual disease.

As shown in **Figure 2**, the median OS of stage IVA and IVB patients were 35 and 31 months, respectively, and no significant difference (*p*=0.582). The median OS of patients with pleural effusion, parenchymal metastases, and extra-abdominal lymph nodes were 35, 30, and 31 months, respectively, also no significant difference among three different metastasis patterns (*p*=0.820, **Figure 3**). Nevertheless, the median OS of cases with abdominal R0 was better than that of those with R1/R2 (74 vs 27 months, *p*=0.000, **Figure 4**), regardless of IVA and IVB stages or metastatic patterns.

The most common site of the first recurrence in stage IV patients with different metastatic diseases was intra-abdominal. In 22 cases with pleural effusions, 5 (26.3%) of 19 cases with pleural effusions had their first recurrence in the pleura alone, compared with 12 (63.2%) of 19 cases in the abdomen; in 49 cases with extra-abdominal lymph nodes, 9 (27.3%) of 33 cases had their first recurrence in extra-abdominal lymph nodes whilst 22 (66.7%) in the abdomen; Of the 62 cases with parenchymal metastases,12 (28.6%) of 42 cases in their first recurrence was a parenchymal metastasis compared to 25 (59.5%) of 42 cases in the abdomen.

Univariate analysis revealed that chemotherapy alone, large ascites volume (≥500 mL), and postoperative macroscopic residual disease, CA125≥500 U/mL were significantly correlated with worse prognostic factors for OS (*p*<0.05), but FIGO stage IVA/IVB or the pattern of metastasis and ≤4 or >4 NACT cycles for IDS were not (**Table 2**). In multivariate analysis, the post-operative macroscopic residual disease remained independently associated with worse OS in EOC patients with FIGO stage IV(*p*=0.001) (**Table 2**).

**Table 2:** Univariate and multivariableanalysisfor OS in EOC patients with FIGO stage IV.

Variables	Univariable analysis		Multivariable analysis	
	HR (95% CI)	*p*-value	HR (95% CI)	*p*-value
Age (years)				
≤60	1			
>60	0.926(0.502-1.706)	0.804		
FIGO stage				
IVA	1			
IVB	0.839(0.446-1.578)	0.586		
Patterns of metastasis				
Pleural effusion	1			
Extra‐abdominal lymph node metastases	0.882(0.436-1.784)	0.727		
Parenchymal metastases	0.809(0.411-1.591)	0.539		
CA125 (U/mL)				
<500	1		1	
≥500	2.225(1.050-4.715)	0.037	1.698(0.774-3.722)	0.186
Ascites				
<500ml	1		1	
≥500 mL	1.875(1.134-3.100)	0.014	1.544(0.914-2.609)	0.104
Treatment				
Chemotherapy only	1		1	
Cytoreductive surgery	0.292 (0.147-0.580)	0.000	0.353(0.176-0.708)	0.003
IDS	1			
PDS	0.865(0.473-1.712)	0.676		
or R0	1		1	
R1/R2	2.991(1.643-5.444)	0.000	2.751(1.486-5.094)	0.001
Histologic type				
High grade serous	1			
Other	0.988(0.358-2.731)	0.982		
NACT cycles (IDS)				
≤4	1			
>4	1.472(0.730-2.969)	0.280		

HR (95% CI), hazard ratio (95% confidence interval); RD, residual disease; R0, complete intra-abdominal tumor resection; R1, residual tumor >0 and ≤1 cm); R2, residual tumor >1 cm); IDS, interval debulking surgery; PDS, Primary debulking surgery; NACT, neoadjuvant chemotherapy.

## Discussion

The most important independent prognostic factor in EOC is the residual disease after cytoreductive surgery. It remains unclear whether patients with stage IV should undergo PDS or NACT-IDS. Also, if postoperative residual disease is a prognostic factor stage IV EOC remains uncertain because of less study addressing this issue. Our study found that the patients for PDS had better OS compared to those for NACT–IDS (43 months vs 31 months), though this was not statistically significant (*p*=0.676) (**Figure 1**). However, it should be noted that our study is a retrospective study with a small sample size, and the results need to be further verified by large prospective control studies. Our study also showed that complete intra-abdominal tumor resection (R0) could significantly improve OS (**Figure 4**) in the patients with stage IV EOC, consistent with previous studies (14, 15). Additionally, it has been reported that for stage IV patients undergoing complete removal of intrahepatic metastases, or even unresectable liver metastases and postoperative residual disease, optimal debulking of extrahepatic disease prolonged their survival and benefited from the reduction surgery (16). As shown in **Figure 1**, our findings showed that cytoreductive surgery PDS or NACT-IDS but not chemotherapy alone could improve the survival of stage IV patients.

The four prospective randomized controlled studies published since 2010 showed that there was no significant difference in PFS and OS in either stage IIIc or IV ovarian cancer patients who underwent PDS or NACT-IDS. The optimal cytoreduction rate was greater with NACT-IDS, and there were fewer postoperative complications and lower perioperative mortality (17–20). The pooled analysis of the results of the European EORTC 55917 and UK CHORUS studies suggested that giving NACT to high-risk patients (stage IV or IIIC with high tumor load) had a survival benefit (9). However, stage IV illness patient data are rare, and many publications typically combine analysis with stage IIIC patient data (10, 11, 16).. The initial treatment of advanced ovarian cancer is still controversial. A study found that women who received NACT had decreased OS compared to those who had PDS in the younger group, stage III disease, and lesser disease extent cases (21). Thus, large prospective population-based studies comparing PDS and IDS in stage IV should be done.

As we know, debulking intra-abdominal tumor disease to no macroscopic visible residual diseases is the most important prognostic factor. Several studies indicated that the number of neoadjuvant chemotherapy cycles did not seem to affect OS in Stage IIIC or IV patients adopting NACT-IDS (12, 22, 23). Other studies, however, found that patients who had >4 cycles of NACT had a poor prognosis regardless of complete resection or a reduced rate of complete cytoreduction (24, 25). One multi-center retrospective study observed that advanced EOC patients receiving ≤4 cycles of NACT had better outcome compared to those patients receiving >5 cycles of NACT (26). Our study showed that the median OS of patients receiving ≤4 NACT followed by early IDS was better than that of those receiving >4 cycles NACT followed by delayed IDS (33 vs 26 months), though this was not statistically significant (*p*=0.272) (**Table 2**). The recurrence of platinum resistance in NACT-IDS was significantly higher than that in PDS *(P*=0.016) which suggests that the primary chemotherapy-sensitive tumor cells could enrich more chemo-resistant clones after >4 cycles of NACT, resulting in a higher incidence of non-surgically detectable minimal residual diseases (27, 28). Previous studies have shown that platinum resistance recurrence (~88%) occurs in patients with advanced EOC for IDS following NACT (29, 30).

The FIGO staging system revised to sub-classification stage IV EOC into IVA and IVB disease according to different metastatic patterns or localizations in 2014 (4), however, there has been considerable debate and concern about whether this sub-classification affects the prognosis of stage IV EOC. Several studies reported that there was no prognostic advantage for pleural metastases only as compared to other stage IV disease manifestations (31–33). Nonetheless, the other study reported that stage IV patients with pleural effusion (stage IVA) displayed a survival advantage compared with other extra-abdominal diseases or parenchymal metastases (34). In addition, patients with inguinal lymph node metastasis and transmural bowel infiltration with mucosal involvement are now considered FIGO stage IVB. How does the prognosis of patients with solely transmural bowel infiltration differ from those of patients with distant extra-abdominal metastasis (e.g., lung, bone, breast, diaphragmatic angle, mediastinum, axillary, or cervical lymph nodes)? There is also a controversial discussion about whether patients with abdominal wall metastases, including puncture hole transfer metastases, should more appropriately be classified as stage IIIC than IVB. It has been reported that the prognosis of these patients seems to be better than that of those patients with distant extra-abdominal metastasis (35). In our study, neither the sub-classification into FIGO IVA and IVB nor metastatic patterns of stage IV EOC provided meaningful prognostic information (**Table 2**, **Figures 2**, **3**).

The most common site of first or any recurrence in all three groups was the abdomen with only a small number of first recurrences arising in their extra-abdominal sites. Several studies showed that most recurrences occur intra-abdominally, which underlines the importance of controlling intra-abdominal disease (34, 36–38). We also observed the most common extra-abdominal site of recurrence was the initial site of stage IV disease, distant metastases seldom occur in absence of intra-abdominal disease and most of them died of progressive abdominal disease. In addition, the subgroup analysis of the SOLO1 study showed that patients with residual lesions after surgery had a reduced risk of disease progression or death by 56% (HR=0.44, 95% CI 0.25-0.77), and patients without residual lesions after surgery had a reduced risk of disease progression or death by 67% (HR=0.33, 95% CI 0.23-0.46) (39), suggesting that complete intra-abdominal tumor resection (R0) followed by first-line PARP inhibitor maintenance therapy would benefit ovarian cancer patient in survival. As suggested by several reports (40–42), the initial survival advantage among EOC patients with *BRCA* mutations may reflect a higher initial sensitivity of *BRCA* carriers to chemotherapy and short-term survival but this response does not predict long-term survival. The strongest predictor of long-term survival is status of no residual disease at resection. EOC patients with *BRCA* mutation were less likely to achieve a state of no residual disease than those without *BRCA* mutation and this difference was not statistically significant in advanced stage serous tumors. Women with no residual disease following PDS or IDS experience significantly superior survival compared to those with any residual disease (43–46). Thus, Irrespective of *BRCA* status, advanced-stage HGSOC patients have a superior prognosis with complete surgical cytoreduction and good histopathological response to chemotherapy. Therefore, surgical control of abdominal disease is still recognized as the most important measure in all stage IV diseases.

Women with stage IV ovarian cancer often have extensive extra-abdominal and intra-abdominal disease; thus, the feasibility and efficacy of primary debulking or ultra-radical surgery have been questioned in the past. Whether intraoperative and postoperative complications associated with complex procedure compromise the survival benefits of the procedure itself remains controversial. There is still no consensus on the optimal therapeutic strategies for patients with FIGO stage IV EOC. Previous studies have shown that more extensive surgery improves cytoreduction rates and survival, however these analyses have primarily focused on stage III patients with a limited proportion of stage IV patients, and contradictory results have also been presented. In our study, isolated extra-abdominal lymph nodes that could be totally resected were removed during surgery, and it was uncertain whether this surgical procedure may increase survival. R0 cytoreductive surgery (CRS) is the most favorable determinant for the prognosis of OC patients, and R0 liver resection (LR) is a component of R0 CRS. Studies have identified the feasibility of surgery to remove metastatic hepatocellular lymph nodes (HCLNs). There is currently a lack of knowledge on the metastatic incidence of HCLNs and how it affects the prognosis of OC. Valerio Gal lotta et al. found that metastatic HCLN status as having an independent, unfavorable prognostic role for PFS, but there had no evidence indicated that HCLN resection might confer a survival advantage for advanced OC patients (47). As the same, though evidence confirmed the safety of cardiophrenic angle lymph node (CPLN) dissection, the correlation between this procedure and prognosis remains unclear. Several studies had found that CPLN dissection did not prove a statistically significant therapeutic benefit (48, 49). Up to date, the impact of HCLN and CPLN resection on survival remains uncertain, and no recommendation can be made based on evidence. So, more studies, especially prospective ones, are needed to evaluate the survival effects of maximal surgical effort among stage IV women and assess the role of HCLN and CPLN resection in advanced ovarian cancer.

Our study confirms that regardless of FIGO IVA and IVB stages or metastatic patterns, patients with stage IV EOC could benefit from cytoreductive surgery with abdominal R0, compared with chemotherapy alone. Surgical management of abdominal disease is still the most important measure in all stage IV diseases. Notable limitations of our study include its retrospective nature and small sample size. Another limitation is that histological diagnosis was not performed on all patients to confirm extra-abdominal lymph node involvement. The third limitation is that the number of people underwent genetic testing and maintenance therapy was few, all patients only underwent laparotomy and intravenous chemotherapy, so we did not include these influencing factors in the analysis. Future studies with larger sample size and more rigorous research designs will help to identify prognostic factors of stage IV EOC patients.

## Conclusion

Patients with stage IV EOC may benefit from cytoreductive surgery with the best abdominal resection irrespective of PDS or NACT-IDS. The prognosis survival of stage IV patients could not be impacted by the sub-classification of IVA and IVB or metastatic patterns. The number of NACT cycles for IDS depends on whether the optimal abdominal resection can be accomplished.

## Data availability statement

The raw data supporting the conclusions of this article will be made available by the authors, without undue reservation.

## Ethics statement

Ethical review and approval was not required for the study on human participants in accordance with the local legislation and institutional requirements. Written informed consent for participation was not required for this study in accordance with the national legislation and the institutional requirements.

## Author contributions

HL: conceptualization, methodology, writing - original draft. ML: statistical analysis, investigation. CP: investigation, data curation and follow-up work. JMeH: statistical analysis. DW: quality control of data and algorithms. JMiH: drafting of the manuscript, writing-reviewing and editing. GZ: conception, design, writing-reviewing and editing. All authors contributed to the article and approved the submitted version.

## References

[1] Sung H Ferlay J Siegel RL Laversanne M Soerjomataram I Jemal A. Global cancer statistics 2020: GLOBOCAN estimates of incidence and mortality worldwide for 36 cancers in 185 countries. CA Cancer J Clin (2021) 71(3):209–49. doi: 10.3322/caac.21660 33538338

[2] Prat J FIGO Committee on Gynecologic Oncology. FIGO's staging classification for cancer of the ovary, fallopian tube, and peritoneum: abridged republication. J Gynecol Oncol (2015) 26(2):87–9. doi: 10.3802/jgo.2015.26.2.87 PMC4397237 25872889

[3] Ataseven B Chiva LM Harter P Gonzalez-Martin A du Bois A. FIGO stage IV epithelial ovarian, fallopian tube and peritoneal cancer revisited. Gynecol Oncol (2016) 142(3):597–607. doi: 10.1016/j.ygyno.2016.06.013 27335253

[4] Ataseven B Harter P Grimm C Heitz F Heikaus S Traut A. The revised 2014 FIGO staging system for epithelial ovarian cancer: Is a subclassification into FIGO stage IVA and IVB justified? Gynecol Oncol (2016) 142(2):243–47. doi: 10.1016/j.ygyno.2016.05.021 27208538

[5] Ataseven B Grimm C Harter P Heitz F Traut A Prader S. Prognostic impact of debulking surgery and residual tumor in patients with epithelial ovarian cancer FIGO stage IV. Gynecol Oncol (2016) 140(2):215–20. doi: 10.1016/j.ygyno.2015.12.007 26691222

[6] Tajik P van de Vrie R Zafarmand MH Coens C Buist MR Vergote I. The FIGO stage IVA versus IVB of ovarian cancer: prognostic value and predictive value for neoadjuvant chemotherapy. Int J Gynecol Cancer (2018) 28(3):453–58. doi: 10.1097/IGC.0000000000001186 29324537

[7] Bonnefoi H A'Hern RP Fisher C Macfarlane V Barton D Blake P. Natural history of stage IV epithelial ovarian cancer. J Clin Oncol (1999) 17(3):767–75. doi: 10.1200/JCO.1999.17.3.767 10071265

[8] Gill SE McGree ME Weaver AL Cliby WA Langstraat CL. Optimizing the treatment of ovarian cancer: Neoadjuvant chemotherapy and interval debulking versus primary debulking surgery for epithelial ovarian cancers likely to have suboptimal resection. Gynecol Oncol (2017) 144(2):266–73. doi: 10.1016/j.ygyno.2016.11.021 27916269

[9] Vergote I Coens C Nankivell M Kristensen GB Parmar MKB Ehlen T. Neoadjuvant chemotherapy versus debulking surgery in advanced tubo-ovarian cancers: pooled analysis of individual patient data from the EORTC 55971 and CHORUS trials. Lancet Oncol (2018) 19(12):1680–7. doi: 10.1016/S1470-2045(18)30566-7 30413383

[10] Curtin JP Malik R Venkatraman ES Barakat RR Hoskins WJ. Stage IV ovarian cancer: impact of surgical debulking. Gynecol Oncol (1997) 64(1):9–12. doi: 10.1006/gyno.1996.4550 8995540

[11] Rauh-Hain JA Rodriguez N Growdon WB Goodman AK Boruta DM 2nd Horowitz NS. Primary debulking surgery versus neoadjuvant chemotherapy in stage IV ovarian cancer. Ann Surg Oncol (2012) 19(3):959–65. doi: 10.1245/s10434-011-2100-x 21994038

[12] Yoneoka Y Ishikawa M Uehara T Shimizu H Uno M Murakami T. Treatment strategies for patients with advanced ovarian cancer undergoing neoadjuvant chemotherapy: interval debulking surgery or additional chemotherapy? J Gynecol Oncol (2019) 30(5):e81. doi: 10.3802/jgo.2019.30.e81 31328461 PMC6658596

[13] Suidan RS Ramirez PT Sarasohn DM Teitcher JB Iyer RB Zhou Q. A multicenter assessment of the ability of preoperative computed tomography scan and CA-125 to predict gross residual disease at primary debulking for advanced epithelial ovarian cancer. Gynecol Oncol (2017) 145(1):27–31. doi: 10.1016/j.ygyno.2017.02.020 28209497 PMC5387995

[14] Aletti GD Dowdy SC Podratz KC Cliby WA. Analysis of factors impacting operability in stage IV ovarian cancer: rationale use of a triage system. Gynecol Oncol (2007) 105(1):84–9. doi: 10.1016/j.ygyno.2006.10.055 17157903

[15] Winter WE 3rd Maxwell GL Tian C Sundborg MJ Rose GS Rose PG. Tumor residual after surgical cytoreduction in prediction of clinical outcome in stage IV epithelial ovarian cancer: a Gynecologic Oncology Group Study. J Clin Oncol (2008) 26(1):83–9. doi: 10.1200/JCO.2007.13.1953 18025437

[16] Bristow RE Montz FJ Lagasse LD Leuchter RS Karlan BY. Survival impact of surgical cytoreduction in stage IV epithelial ovarian cancer. Gynecol Oncol (1999) 72(3):278–87. doi: 10.1006/gyno.1998.5145 10053096

[17] Vergote I Tropé CG Amant F Kristensen GB Ehlen T Johnson N. Neoadjuvant chemotherapy or primary surgery in stage IIIC or IV ovarian cancer. N Engl J Med (2010) 363(10):943–53. doi: 10.1056/NEJMoa0908806 20818904

[18] Kehoe S Hook J Nankivell M Jayson GC Kitchener H Lopes T. Primary chemotherapy versus primary surgery for newly diagnosed advanced ovarian cancer (CHORUS): an open-label, randomised, controlled, non-inferiority trial. Lancet (2015) 386(9990):249–57. doi: 10.1016/S0140-6736(14)62223-6 26002111

[19] Fagotti A Ferrandina G Vizzielli G Fanfani F Gallotta V Chiantera V. Randomized trial of primary debulking surgery versus neoadjuvant chemotherapy for advanced epithelial ovarian cancer (SCORPION-NCT01461850). Int J Gynecol Cancer (2020) 30:1657–64. doi: 10.1136/IJGC-2020-001640 33028623

[20] Onda T Satoh T Saito T Kasamatsu T Nakanishi T Nakamura K. Comparison of treatment invasiveness between upfront debulking surgery versus interval debulking surgery following neoadjuvant chemotherapy for stage III/IV ovarian, tubal, and peritoneal cancers in a phase III randomised trial: Japan Clinical Oncology Group Study JCOG0602. Eur J Cancer (2016) 64:22–31. doi: 10.1016/j.ejca.2016.05.017 27323348

[21] Matsuo K Matsuzaki S Nusbaum DJ Maoz A Oda K Klar M. Possible candidate population for neoadjuvant chemotherapy in women with advanced ovarian cancer. Gynecol Oncol (2021) 160(1):32–9. doi: 10.1016/j.ygyno.2020.10.027 33196436

[22] Akladios C Baldauf JJ Marchal F Hummel M Rebstock LE Kurtz JE. Does the number of neoadjuvant chemotherapy cycles before interval debulking surgery influence survival in advanced ovarian cancer? Oncology (2016) 91(6):331–40. doi: 10.1159/000449203 27784027

[23] Betrian S Angeles MA Gil Moreno A Cabarrou B Deslandres M Ferron G. Survival impact of histological response to neoadjuvant chemotherapy according to number of cycles in patients with advanced ovarian cancer. Int J Gynecol Cancer (2022) 32:967–74. doi: 10.1136/ijgc-2021-003313 35858711

[24] Colombo PE Labaki M Fabbro M Bertrand M Mourregot A Gutowski M. Impact of neoadjuvant chemotherapy cycles prior to interval surgery in patients with advanced epithelial ovarian cancer. Gynecol Oncol (2014) 135(2):223–30. doi: 10.1016/j.ygyno.2014.09.002 25220627

[25] Phillips A Sundar S Singh K Nevin J Elattar A Kehoe S. Complete cytoreduction after five or more cycles of neo-adjuvant chemotherapy confers a survival benefit in advanced ovarian cancer. Eur J Surg Oncol (2018) 44(6):760–5. doi: 10.1016/j.ejso.2018.01.097 29426779

[26] Lecointre L Velten M Lodi M Saadeh R Lavoué V Ouldamer L. Impact of neoadjuvant chemotherapy cycles on survival of patients with advanced ovarian cancer: A French national multicenter study (FRANCOGYN). Eur J Obstet Gynecol Reprod Biol (2020) 245:64–72. doi: 10.1016/j.ejogrb.2019.12.001 31864157

[27] Lim MC Song YJ Seo SS Yoo CW Kang S Park SY. Residual cancer stem cells after interval cytoreductive surgery following neoadjuvant chemotherapy could result in poor treatment outcomes for ovarian cancer. Onkologie (2010) 33(6):324–30. doi: 10.1159/000313823 20523098

[28] Himoto Y Cybulska P Shitano F Sala E Zheng J Capanu M. Does the method of primary treatment affect the pattern of first recurrence in high-grade serous ovarian cancer? Gynecol Oncol (2019) 155(2):192–200. doi: 10.1016/j.ygyno.2019.08.011 31521322 PMC6837278

[29] Zhang GN Liu H Huang JM Wang L Zhao JS Li C. TP53 K351N mutation-associated platinum resistance after neoadjuvant chemotherapy in patients with advanced ovarian cancer. Gynecol Oncol (2014) 132(3):752–57. doi: 10.1016/j.ygyno.2014.01.028 24463159

[30] Rauh-Hain JA Nitschmann CC Worley MJ Jr. Bradford LS Berkowitz RS Schorge JO. Platinum resistance after neoadjuvant chemotherapy compared to primary surgery in patients with advanced epithelial ovarian carcinoma. Gynecol Oncol (2013) 129(1):63–8. doi: 10.1016/j.ygyno.2013.01.009 23337490

[31] Paik ES Lee YY Lee EJ Choi CH Kim TJ Lee JW. Survival analysis of revised 2013 FIGO staging classification of epithelial ovarian cancer and comparison with previous FIGO staging classification. Obstet Gynecol Sci (2015) 58(2):124–34. doi: 10.5468/ogs.2015.58.2.124 PMC4366865 25798426

[32] Rosendahl M Høgdall CK Mosgaard BJ. Restaging and survival analysis of 4036 ovarian cancer patients according to the 2013 FIGO classification for ovarian, fallopian tube, and primary peritoneal cancer. Int J Gynecol Cancer (2016) 26(4):680–7. doi: 10.1097/IGC.0000000000000675 26937751

[33] Hjerpe E Staf C Dahm-Kähler P Stålberg K Bjurberg M Holmberg E. Lymph node metastases as only qualifier for stage IV serous ovarian cancer confers longer survival than other sites of distant disease - a Swedish Gynecologic Cancer Group (SweGCG) study. Acta Oncol (2018) 57(3):331–7. doi: 10.1080/0284186X.2017.1400691 29130381

[34] Aletti GD Podratz KC Cliby WA Gostout BS. Stage IV ovarian cancer: disease site-specific rationale for postoperative treatment. Gynecol Oncol (2009) 112(1):22–7. doi: 10.1016/j.ygyno.2008.09.010 18947860

[35] Prat J. Staging classification for cancer of the ovary, fallopian tube, and peritoneum. Int J Gynaecol Obstet (2014) 124(1):1–5. doi: 10.1016/j.ijgo.2013.10.001 24219974

[36] Perri T Ben-Baruch G Kalfon S Beiner ME Helpman L Hogen LB. Abdominopelvic cytoreduction rates and recurrence sites in stage IV ovarian cancer: is there a case for thoracic cytoreduction? Gynecol Oncol (2013) 131(1):27–31. doi: 10.1016/j.ygyno.2013.07.093 23880152

[37] Jamieson A Sykes P Eva L Bergzoll C Simcock B. Subtypes of stage IV ovarian cancer; response to treatment and patterns of disease recurrence. Gynecol Oncol (2017) 146(2):273–8. doi: 10.1016/j.ygyno.2017.05.023 28549816

[38] Timmermans M Sonke GS Van de Vijver KK Ottevanger PB Nijman HW van der Aa MA. Localization of distant metastases defines prognosis and treatment efficacy in patients with FIGO stage IV ovarian cancer. Int J Gynecol Cancer (2019) 29(2):392–7. doi: 10.1136/ijgc-2018-000100 30665898

[39] Moore K Colombo N Scambia G Kim BG Oaknin A Friedlander M. Maintenance olaparib in patients with newly diagnosed advanced ovarian cancer. N Engl J Med (2018) 379(26):2495–505. doi: 10.1056/NEJMoa1810858 30345884

[40] Kotsopoulos J Rosen B Fan I Moody J McLaughlin JR Risch H. Ten-year survival after epithelial ovarian cancer is not associated with BRCA mutation status. Gynecol Oncol (2016) 140(1):42–7. doi: 10.1016/j.ygyno.2015.11.009 26556769

[41] De Jong D Otify M Chen I Jackson D Jayasinghe K Nugent D. Survival and Chemosensitivity in Advanced High Grade Serous Epithelial Ovarian Cancer Patients with and without a BRCA Germline Mutation: More Evidence for Shifting the Paradigm towards Complete Surgical Cytoreduction. Medicina (Kaunas) (2022) 58(11):1611. doi: 10.3390/medicina58111611 36363568 PMC9699274

[42] Petrillo M Marchetti C De Leo R Musella A Capoluongo E Paris I. BRCA mutational status, initial disease presentation, and clinical outcome in high-grade serous advanced ovarian cancer: a multicenter study. Am J Obstet Gynecol (2017) 217(3):334.e1–9. doi: 10.1016/j.ajog.2017.05.036 28549976

[43] Rosen B Laframboise S Ferguson S Dodge J Bernardini M Murphy J. The impacts of neoadjuvant chemotherapy and of debulking surgery on survival from advanced ovarian cancer. Gynecol Oncol (2014) 134(3):462–7. doi: 10.1016/j.ygyno.2014.07.004 25026637

[44] Kim JY Park H Lee DW Kim MJ Shin JE Lee KE. Prognostic significance of clinical factors including BRCA mutation in epithelial ovarian, peritoneal, fallopian tube cancer. Anticancer Res (2022) 42(10):4945–54. doi: 10.21873/anticanres.16001 36191979

[45] Aletti GD Dowdy SC Gostout BS Jones MB Stanhope CR Wilson TO. Aggressive surgical effort and improved survival in advanced-stage ovarian cancer. Obstet Gynecol (2006) 107(1):77–85. doi: 10.1097/01.AOG.0000192407.04428.bb 16394043

[46] Kim SR Malcolmson J Li X Bernardini MQ Hogen L May T. The correlation between BRCA status and surgical cytoreduction in high-grade serous ovarian carcinoma. Gynecol Oncol (2021) 162(3):702–6. doi: 10.1016/j.ygyno.2021.07.010 34256977

[47] Gallotta V Ferrandina G Vizzielli G Conte C Lucidi A Costantini B. Hepatoceliac lymph node involvement in advanced ovarian cancer patients: prognostic role and clinical considerations. Ann Surg Oncol (2017) . 24(11):3413–21. doi: 10.1245/s10434-017-6005-1 28718034

[48] Addley S Asher V Kirke R Bali A Abdul S Phillips A. What are the implications of radiologically abnormal cardiophrenic lymph nodes in advanced ovarian cancer? An analysis of tumour burden, surgical complexity, same-site recurrence and overall survival. Eur J Surg Oncol (2022) 48(12):2531–8. doi: 10.1016/j.ejso.2022.06.006 35718677

[49] Prader S Vollmar N du Bois A Heitz F Schneider S Ataseven B. Pattern and impact of metastatic cardiophrenic lymph nodes in advanced epithelial ovarian cancer. Gynecol Oncol (2019) 152(1):76–81. doi: 10.1016/j.ygyno.2018.11.001 30463683

